# Ongoing substrate-driven atrial fibrillation “boxed” in the left atrial posterior wall with ablation: a case report

**DOI:** 10.3389/fcvm.2023.1251874

**Published:** 2023-09-14

**Authors:** Vassil Traykov, Daniel Marchov, Emiliyan Martinov, Asmaa El Abbady, Valeri Gelev, Wolfgang Dichtl

**Affiliations:** ^1^ Clinic of Cardiology, Acibadem City Clinic Tokuda Hospital, Sofia, Bulgaria; ^2^ Cardiac Electrophysiology Department, National Heart Institute, Giza, Egypt; ^3^ University Hospital for Internal Medicine III (Cardiology and Angiology), Medical University of Innsbruck, Innsbruck, Austria

**Keywords:** posterior wall, box lesion, independent tachycardia, ablation, case report

## Abstract

Patients undergoing valve surgery for rheumatic heart disease are expected to develop significant atrial arrhythmogenic substrates outside of the pulmonary veins, which sometimes require complex ablation techniques for the treatment of symptomatic arrhythmias. We describe, herein, the case of a 76-year-old male undergoing endocardial ablation for the treatment of symptomatic persistent atrial fibrillation which developed after aortic and mitral valve replacement with a simultaneous tricuspid ring annuloplasty. Following pulmonary vein isolation, the patient's atrial fibrillation was converted into cavotricuspid isthmus-dependent atrial flutter. After a successful cavotricuspid isthmus ablation, the arrhythmia reverted back to a left atrial tachyarrhythmia originating from the posterior wall. A linear left atrial lesion led to the electrical isolation of a large area, which included the posterior wall, as well as the containment of the ongoing fibrillatory activity, while sinus rhythm was restored in the rest of the atria. In conclusion, successful left atrial posterior wall isolation can be achieved in the setting of severe scarring due to previous atriotomy by creating a linear lesion on the atrial roof, in conjunction with pulmonary vein isolation, sparing the patient from requiring bottom-line ablation, and avoiding possible esophageal injury. Such compartmentalization of the left atrium may effectively contain local fibrillatory activity, while allowing for the restoration of sinus rhythm.

## Introduction

Pulmonary vein isolation (PVI) is the mainstay of atrial fibrillation (AF) ablation. At present, it is unclear whether or not the ablation of additional substrates targeting electrical isolation in other atrial areas, such as the left atrium (LA) or left atrial posterior wall (LAPW), provides the same benefit, as no randomized clinical trials have shown improved benefits (1, 2). Macroreentrant atrial arrhythmias can occur late after mitral valve replacement due to the scarring associated with atriotomy. Extensive scarring may also occur in remote areas of the LA, particularly in patients with a history of rheumatic heart disease (3). The case report presented herein describes the case of a patient with a long history of AF, who underwent mitral and aortic valve replacement in conjunction with tricuspid ring annuloplasty for the treatment of rheumatic valvular heart disease. The patient underwent a successful isolation of a large low-voltage area of the LAPW through PVI and the creation of a linear lesion on the roof of the LA, which resulted in the restoration of sinus rhythm (SR) to the rest of the atria, although fibrillatory activity continued in the isolated area.

## Case description

A 76-year-old male was referred to a tertiary arrhythmia center with palpitations and shortness of breath. The patient had a childhood history of numerous bouts of rheumatic fever, which resulted in severe mitral regurgitation and moderate stenosis, along with moderate aortic and severe tricuspid regurgitation. Subsequently, the patient underwent aortic and mitral valve replacement with tissue valves, as well as tricuspid ring annuloplasty, at 71 years of age. Over time, the patient developed AF, which had already been staged as permanent at the time he had the aforementioned surgery (Supplementary Table).

Five years after his valve replacement surgery, the patient presented to his general cardiologist with symptomatic AF (European Heart Rhythm Association class III), which required rate control optimization. Echocardiography demonstrated a severely dilated LA (59 mm diameter, LA volume index calculated at 52 ml/m^2^); although the left ventricular size and ejection fraction were normal, as were the tissue valves and the pulmonary artery systolic pressure, which was estimated to be 43 mmHg. Due to the difficulty in achieving rate control, the patient was fitted with an implanted single-chamber pacemaker for physiological pacing of the left bundle branch area in preparation for an atrioventricular node ablation, which was scheduled for 6 weeks later. Surprisingly, upon admission for the ablation, the patient presented with SR); however, despite the initiation of amiodarone therapy, the patient experienced a recurrence of atrial fibrillation shortly thereafter. A decision involving the patient and heart team was made to pursue rhythm control, and after providing written informed consent, the patient was scheduled for a complex catheter ablation.

**Figure 1:**
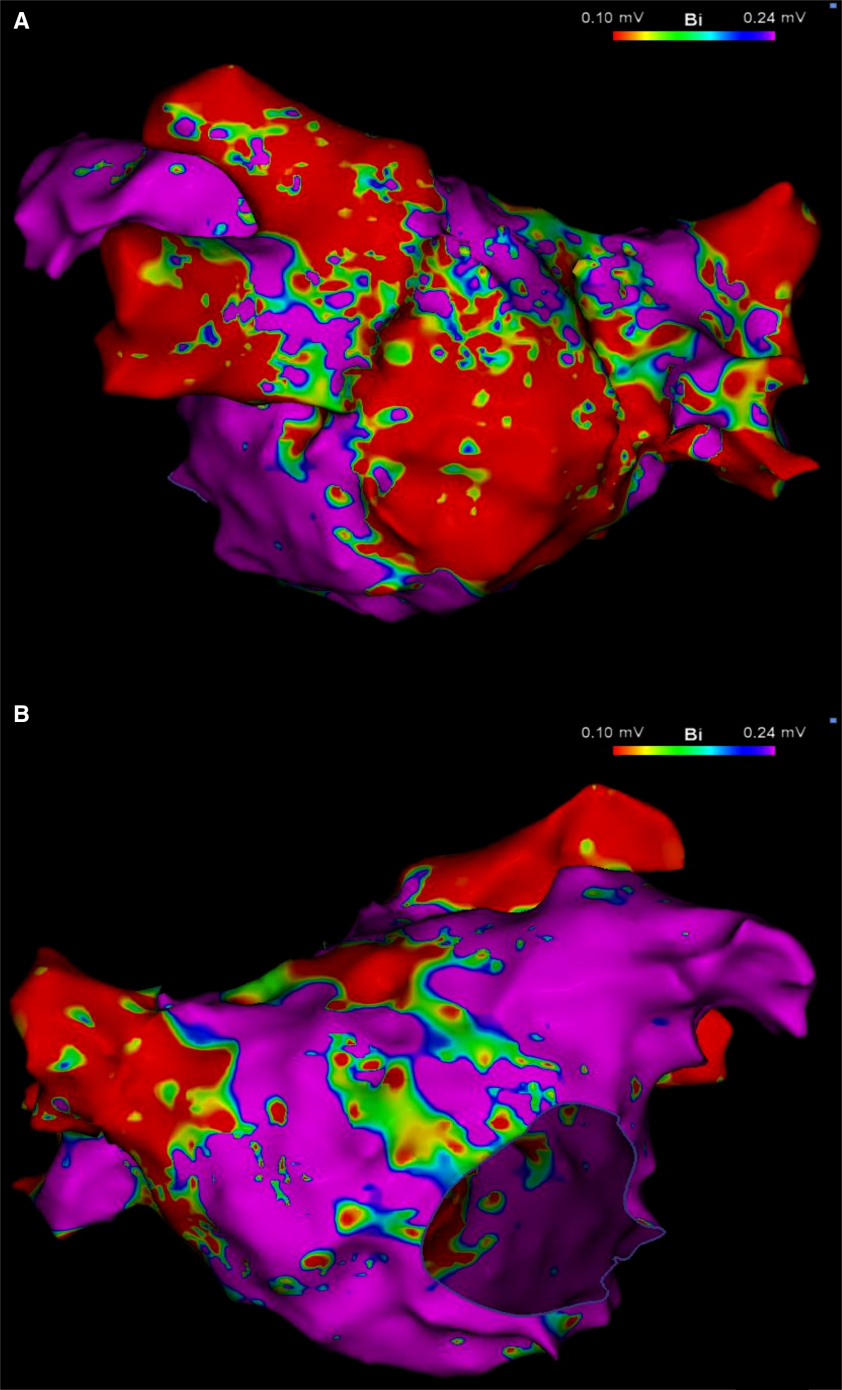
Left atrial voltage map in posteroanterior (**A**) and anteroposterior (**B**) views, created during AF demonstrating a large area of low voltage occupying mainly, but not exclusively, the LAPW. Color bar settings 0.1–0.24 mV as suggested by Rodríguez-Mañero et al. (4). AF, atrial fibrillation; LAPW, left atrial posterior wall.

After securing double transseptal access, a high-density electroanatomical map of the LA was created using a commercially available mapping system (CARTO3; Biosense Webster, Irvine, CA, USA) and a multipolar mapping catheter (PentaRay; Biosense Webster, Irvine, CA, USA). Created while the patient was in AF, the voltage map demonstrated large areas of low voltage, primarily along the posterior wall of the LA, sparing the vestibular portion and the left atrial appendage (Figure 1). Voltage cut-off was 0.24 mV, as suggested by Rodríguez-Mañero et al. (4). PVI was performed with an irrigated-tip catheter (Thermocool Smarttouch SF; Biosense Webster, Irvine, CA, USA) following the CLOSE protocol (5).

**Figure 2:**
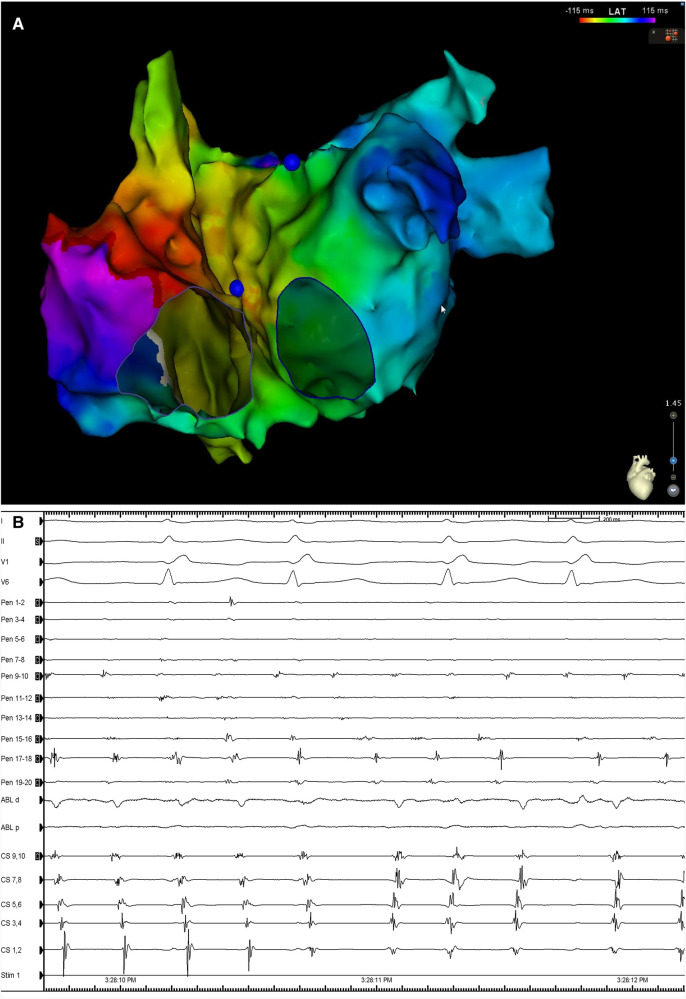
(**A**) Biatrial map during atrial flutter demonstrating the presence of a clockwise peritricuspid re-entry circuit with passively activated LA. (**B**) Surface ECG leads I, II, V1 and V6, along with intracardiac recordings from the multipolar mapping catheter (Pen) located at the LAPW, the ablation catheter (Abl) located at the cavotricuspid isthmus, and a decapolar catheter in the CS. Note the change in the activation in the activation sequence in the CS. Sweep speed was 100 mm/s. LA, left atrium; ECG, electrocardiogram; CS, coronary sinus; other abbreviations as in Figure 1.

**Figure 3:**
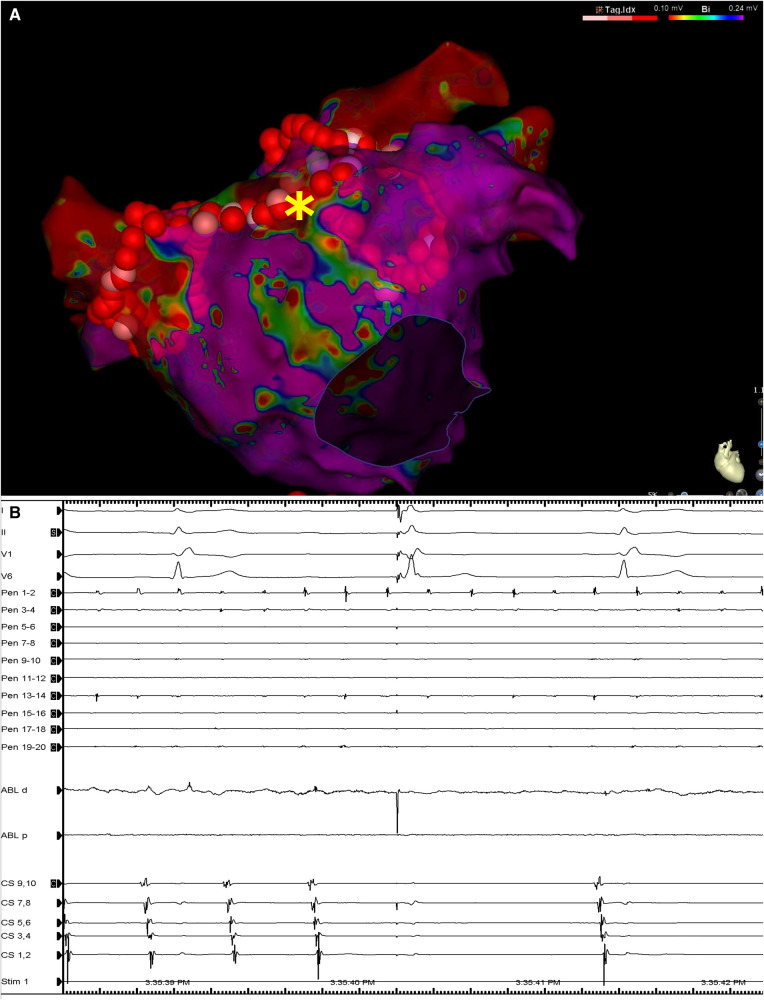
(**A**) Left atrial voltage map in anteroposterior view demonstrating the lesion set including circumferential lesions encircling both PV antra as well as a linear lesion at the LA roof. The asterisk denotes the location at which RF application resulted in LAPW isolation and arrhythmia termination. (**B**) Surface ECG leads I, II, V1 and V6, along with intracardiac recordings from the multipolar mapping catheter located at the LAPW (Pen), the mapping/ablation catheter, and the decapolar catheter in the coronary sinus. Note the termination of arrhythmia in the rest of the atria as seen on the coronary sinus catheter bipoles and ongoing atrial fibrillation/atrial flutter at the LAPW. Sweep speed was 100 mm/s. MAP, mapping/ablation catheter; RF, radiofrequency; other abbreviations as in Figures 1, 2.

During delivery of energy to the LA ridge, the AF was converted into atrial flutter with a cycle length of 240 ms, demonstrating proximal-to-distal coronary sinus activation. Biatrial mapping revealed passive left atrial activation and clockwise peritricuspid atrial flutter circuit (Figure 2A). During ablation at the cavotricuspid isthmus, there was a sudden change in the coronary sinus activation pattern, which reversed to a distal-to-proximal direction, suggesting a left atrial origin (Figure 2B). At the same time, the LAPW was activated with a shorter cycle length, which varied between 200 and 230 ms. Posterior wall isolation was then performed, starting with the roof line. A few lesions on the roof of the LA in the zone of fragmented signals resulted in a progressive delay of conduction from the LAPW to the remaining atria, while SR was eventually restored and the atrial arrhythmia was fully contained in the LAPW (Figure 3). Isolation of the LAPW with persistent atrial arrhythmia and an exit block to the rest of the LA was maintained through the end of the procedure. No attempt at restoration of normal rhythm in the isolated area was made. The isolated area was measured offline, and found to be 72.1 cm^2^, accounting for 28.2% of the surface area of the LA.

The day after the procedure, when he was discharged, the patient was confirmed to be in SR, and no periprocedural complications were observed. Over the course of a 12-month follow-up, the patient remained in SR, and had a significantly improved quality of life. Life-long oral anticoagulation therapy was recommended.

## Discussion

The LAPW stems from the same embryological origin as the pulmonary veins, and the heterogeneous arrangement of its myocardial fibers may result in non-uniform anisotropy with delayed conduction, a unidirectional block, and localized reentry. Compared to other areas of the LA, the cells in the LAPW have larger late sodium currents, more calcium content, and smaller potassium currents, resulting in a low resting membrane potential and short action potential duration (6, 7). In patients who experience rheumatic heart disease following mitral valve surgery, significant scarring at the LAPW, independent from the atriotomy lines, has been described as a marker of atrial cardiomyopathy (8).

**Figure 4:**
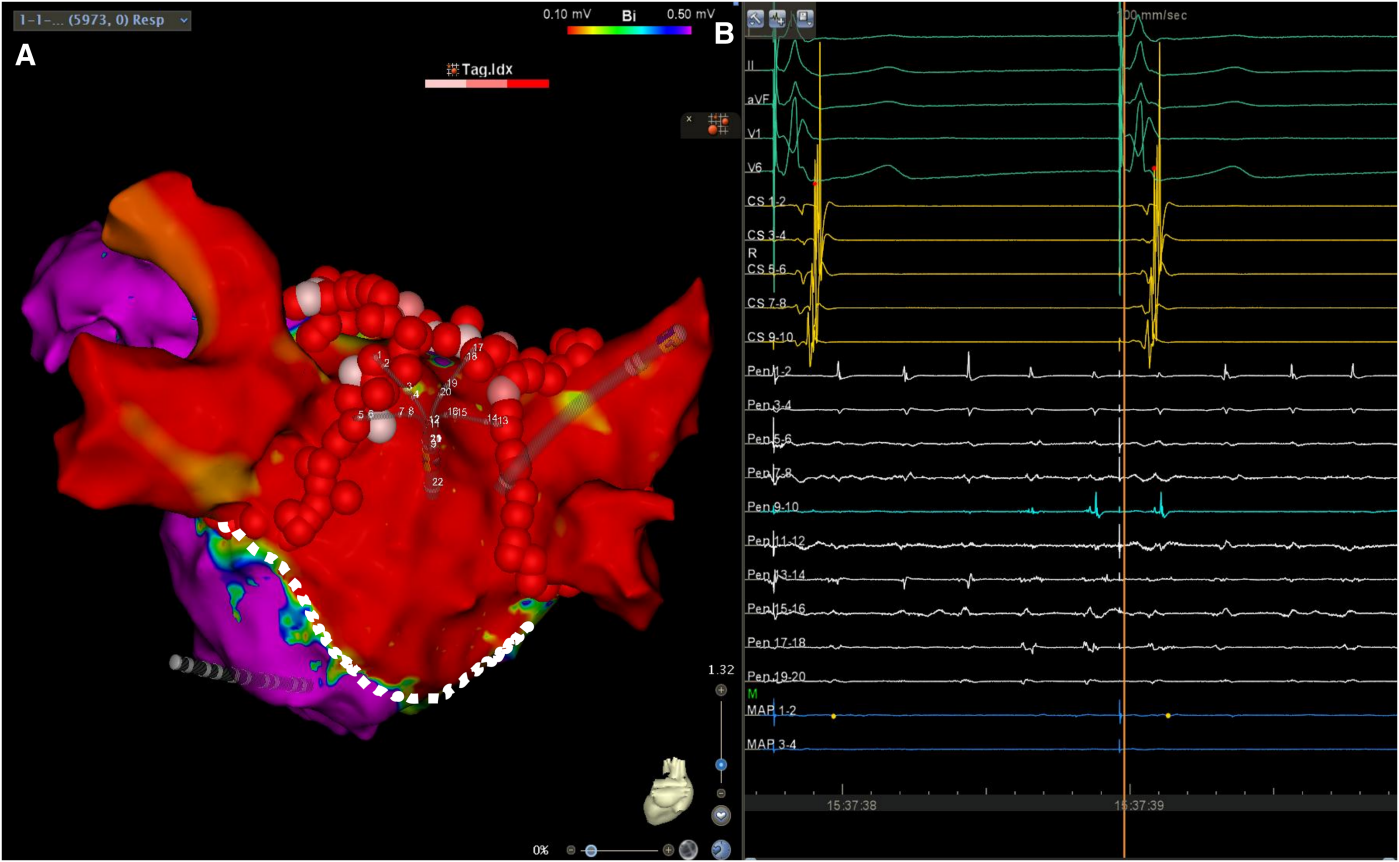
(**A**) Left atrial voltage map in posteroanterior view demonstrating a wide area of low voltage occupying the LAPW. The lesion set consisting of circumferential antral PV isolation and a roof line lesion connecting the upper veins is also shown. The silhouette of the multipolar mapping catheter is shown to be located on the LAPW. (**B**) Surface ECG leads I, II, aVF, V1 and V6, along with intracardiac recordings from the multipolar mapping catheter (Pen) located at the LAPW, the mapping/ablation catheter, and the decapolar catheter in the coronary sinus. Note the ongoing atrial fibrillation/atrial flutter at the LAPW, while the rest of the atrium is activated following retrograde ventriculoatrial conduction by the pacemaker effectively stimulating the conduction system. Sweep speed was 100 mm/s. Sweep speed was 100 mm/s. The dotted line shows the presumed atriotomy line, which most likely aided LAPW isolation using a roof line only. Color bar settings 0.1–0.5 mV. Abbreviations as in Figures 1, 2.

In the case presented herein, LAPW isolation was achieved via PVI and a roof line only, without creating an inferior line, a finding which can be explained by several mechanisms. Using a right lateral approach to the left atrium, atriotomy for mitral valve replacement can be extended to run parallel to the mitral annulus. This, in turn, may explain how point ablation could lead to a delay in conduction from the LAPW across the roof, and why LAPW isolation could be accomplished via a roof line only (Figure 4). An alternative explanation for this would be the presence of a pre-existing functional block at the inferior periphery of the scar, which is primarily due to abrupt changes in myocardial strand orientation (9), scarring by the rheumatic process itself (3), or both. The large surface area of the box created during the ablation, which extended to the anterior wall and atrial septum, could have also contributed to the success of the procedure, due to LA compartmentalization (10).

Similar case reports are scarce, as we found only one relevant case report, describing the final isolation of the posterior LA and restoration of SR with single-point ablation to the roofline gap after an unsuccessful thoracoscopic ablation in a patient with longstanding persistent AF (11).

In conclusion, the case presented herein supports the concept that a large and completely isolated box lesion is the key to a successful ablation in patients with advanced atrial cardiomyopathy due to a history of rheumatic disease. Additionally, the present case report indicated that scarring due to a previous atriotomy affects the ablation strategy.

## Data Availability

The original contributions presented in the study are included in the article/**Supplementary Material**, further inquiries can be directed to the corresponding author.
