# Supplementary material for: Ongoing substrate-driven atrial fibrillation “boxed” in the left atrial posterior wall with ablation: a case report
Source: Front Cardiovasc Med. 2023 Sep 14;10:1251874. doi: 10.3389/fcvm.2023.1251874 (PMC10542893; doi:10.3389/fcvm.2023.1251874)
Supplement: Supplementary file 1 [file Table1.docx]

| Time | Events |
| --- | --- |
| Age of 5 | Rheumatic heart disease |
| Later in life | Diagnosed with combined mitral valve disease and aortic valve disease, atrial fibrillation |
| Age of 71 | Aortic and mitral valve replacement, tricuspid ring annuloplasty, permanent atrial fibrillation accepted |
| Age of 76 | Difficult rate control, single chamber pacemaker implanted (left bundle branch area pacing) in preparation for AV node ablation |
| Six weeks later | Sinus rhythm found to be restored, decision for rhythm control strategy, catheter ablation performed |

Timeline of events in the reported case
